# Differentiating Small (**≤**1 cm) Focal Liver Lesions as Metastases or Cysts by means of Computed Tomography: A Case-Study to Illustrate a Fuzzy Logic-Based Method to Assess the Impact of Diagnostic Confidence on Radiological Diagnosis

**DOI:** 10.1155/2014/587976

**Published:** 2014-01-27

**Authors:** Rossano Girometti, Francesco Fabris, Andrea Sgarro, Gloria Zanella, Serena Pullini, Lorenzo Cereser, Giuseppe Como, Chiara Zuiani, Massimo Bazzocchi

**Affiliations:** ^1^Institute of Diagnostic Radiology, University of Udine Az. Ospedaliero-Universitaria “S. Maria della Misericordia”, Via Colugna No. 50, 33100 Udine, Italy; ^2^Department of Mathematics and Earth Sciences, University of Trieste, Via Valerio 12b, 34127 Trieste, Italy; ^3^Department of Emergency Medicine, Palmanova Hospital, Via Natisone 1, 33057 Palmanova, Italy

## Abstract

*Purpose*. To quantify the impact of diagnostic confidence on radiological diagnosis with a fuzzy logic-based method. *Materials and Methods*. Twenty-two oncologic patients with 20 cysts and 30 metastases ≤1 cm in size found at 64-row computed tomography were included. Two readers (R1/R2) expressed diagnoses as a subjective level of confidence *P*(*d*) in malignancy within the interval [0,1] rather than on a “crisp” basis (malignant/benign); confidence in benignancy was 1 − *p*(*d*). When cross-tabulating data according to the standard of reference, 2 × 2 table cells resulted from the aggregation between *p*(*d*)/1 − *p*(*d*) and final diagnosis. We then assessed (i) readers diagnostic performance on a fuzzy and crisp basis; (ii) the “divergence” *δ*(*F*, *C*) (%) as a measure of how confidence impacted on crisp diagnosis. *Results*. Diagnoses expressed with lower confidence increased fuzzy false positives compared to crisp ones (from 0 to 0.2 for R1; from 1 to 2.4 for R2). Crisp/fuzzy accuracy was 94.0%/93.6% (R1) and 94.0/91.6% (R2). *δ*(*F*, *C*) (%) was larger in the case of the less experienced reader (R2) (up to +7.95% for specificity). According to simulations, *δ*(*F*, *C*) (%) was negative/positive depending on the level of confidence in incorrect diagnoses. *Conclusion.* Fuzzy evaluation shows a measurable effect of uncertainty on radiological diagnoses.

## 1. Introduction

The confidence underlying medical diagnosis has a pivotal impact on clinical decisions [1]. Based on different levels of diagnostic confidence (DC), a specific therapy can be promptly instituted or withheld while waiting for the results of additional investigations. This translates into important consequences in terms of appropriateness, efficacy, and costs of therapies [2]. Similar concepts can be extended to radiological diagnosis. The widespread use of conceptual instruments such as the Breast Imaging Reporting and Data System (BI-RADS) exemplifies the need for taking into account the DC (e.g., expressed as a diagnostic probability) in order to guide patients' management [3].

Quantifying the effect that a diagnostic test has on DC serves as an assessment of the efficacy of that test [4, 5]. To our knowledge, a number of analytic methods have been proposed for this purpose [6], especially in order to take into account the impact of incorrectly confident diagnoses on patients management [2, 7]. In general, these methods assess the changes between pre- and posttest confidences of referring physicians by using proportions or scoring systems (e.g., 1 to 5 scale) rather than a continuum of information. Consequently, these methods (i) do not reflect the variability of DC inherent to test interpretation, and consequently they do not express the radiologist point of view and (ii) do not measure the direct effect of DC levels on diagnostic performance (i.e., the “radiologist efficacy” rather than “test efficacy”). As previously emphasized by Castanho et al. [8] fuzzy logic, which is a cognate to sets theory introduced by Zadeh in 1965 [9], has the potential to contribute to this field. Fuzzy logic is successfully used in many technology systems and has been repeatedly investigated for clinical applications [10]. By definition, this approach is used when it is difficult to classify objects in collections through a binary (“crisp”) process. Accordingly, intermediate membership degrees are defined into the interval [0,1], [11]. For instance, a man with 1000 hairs can be viewed as belonging either to the set of bald men (e.g., with a membership degree of 0.8) or to the set of not bald men (with a membership degree of 0.2). Using the same conceptual framework, a radiological diagnosis (e.g., liver metastasis) might be viewed as belonging to both sets of correct and incorrect diagnoses at the same time, depending on the uncertainty with which the reader achieved it on the basis of the available radiological signs. One (1) or zero (0) values (i.e., the lesion “is” or “is not” a metastasis, resp.) then become a special case of a continuum of (un)certainty rather than absolute values.

To our knowledge, no previous studies compared crisp and fuzzy diagnostic performances. In this study, we assumed that levels of DC in a given diagnosis are equivalent to the fuzzy membership degrees expressing how much that diagnosis belongs to the sets of correct diagnoses. We then adjusted the diagnostic performance of radiologists for the level of DC, based on the method used by Castanho et al. [8], to “fuzzify” sensitivity and specificity (and, by extension, predictive values and accuracy). Crisp and fuzzy diagnostic performances of radiologists were compared accordingly, introducing a measure that we named “divergence.” The method was tested in a simplified, dichotomous clinical scenario, in order to verify whether the effect of DC on diagnostic performance is measurable on real readers. Additionally, we provided two simulations from clinical data in order to emphasize the effect of confidently incorrect diagnoses on diagnostic accuracy.

## 2. Materials and Methods

Because of the retrospective design and the theoretical nature of the study (leading to the absence of clinical implications for patients), the approval by Institutional Review Board was not required, according to laws and regulations of our country. However, patients' data were managed according to the ethical principles for medical research as stated by the Declaration of Helsinki, and patients gave informed consent to undergo computed tomography (CT).

### 2.1. Clinical Model and Patients Population

**Figure 1:**
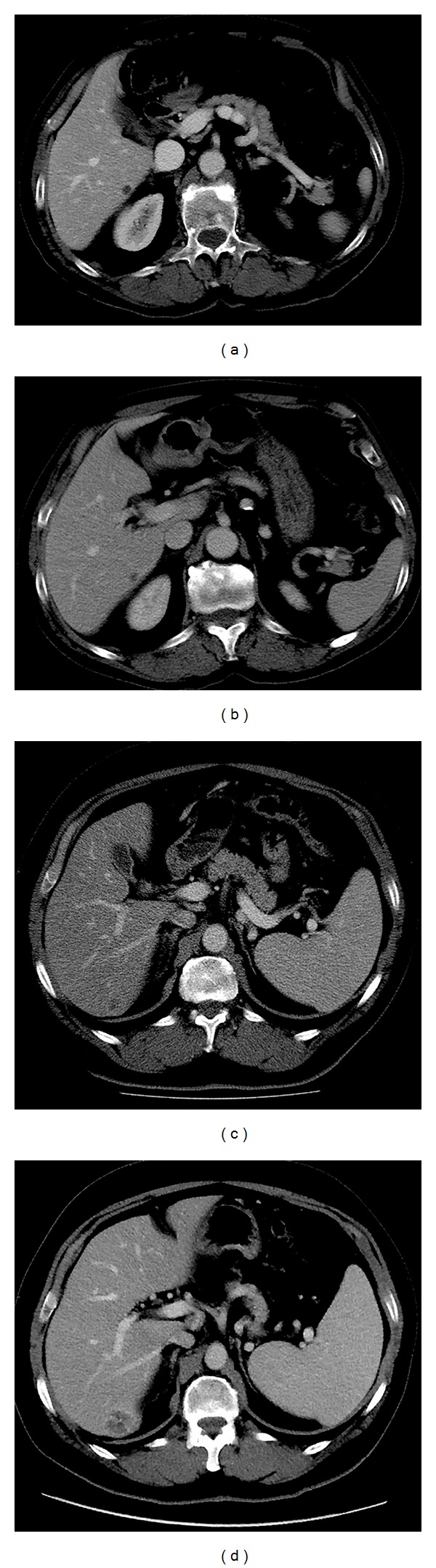
Readers assessed small (≤1 cm) focal liver lesions as cyst or metastasis on 64-row Computed Tomography (CT) axial images acquired in the venous phase. Lesions were confirmed to be cysts (a) when showing no changes (b) at imaging followup, as occurred in this 71-year-old male patient with prostate cancer. On the contrary, lesions (c) showing increase in size during the followup (d) were assessed as metastases, as occurred in this 57-year-old male patient operated for colonic cancer.

We searched in our institutional database for all oncologic patients who performed a baseline abdominal or thoracoabdominal CT between March 2009 and March 2010. Of them, we included those presenting up to five focal livers lesions matching the following criteria: (i) a maximum diameter ≤1 cm on axial images; (ii) hypodense appearance on venous and/or equilibrium phases as compared to the surrounding liver; (iii) to have been assessed as metastases or cysts by a consensus panel of two experienced radiologists who reviewed images of baseline and follow-up CTs, magnetic resonance imaging (MRI), and/or ultrasonography (US) examinations. They assessed as metastases those lesions showing any modification in dimensions and attenuation characteristics at CT, whereas lesions remaining stable were assessed as cysts (Figure 1). Alternatively, they assessed the lesions based on their appearance at MRI or US. Additional inclusion criteria were represented by the absence of extrahepatic findings in the upper abdomen scan. Excluded were patients who did not match the above criteria, including those with additional focal liver lesions of any nature.

Final population was represented by 22 patients (8 male and 14 female; age range 30–79, mean 63.6), who presented 50 focal liver lesions (range 1–5, mean 1.9). According to the standard of reference (range of the follow-up time 12–24 months; mean 12.6 months), 30 lesions were metastases and 20 cysts (per-patient number of follow-up CTs ranged 2–9, mean 4). Of lesions, 5/30 metastases and 2/20 cysts had additional confirmation at subsequent MRI and/or US examination. Primary site of metastases was breast cancer (*n* = 5), colonic cancer (*n* = 16), cholangiocarcinoma (*n* = 3), pancreatic cancer (*n* = 3), lung cancer (*n* = 2), and melanoma (*n* = 1).

### 2.2. CT Protocol

Patients underwent examinations on multidetector CT scanner (LightSpeed CT750 HD, GE Healthcare, Milwaukee, WI, USA) with 64 sections at a detector collimation of 0.625 mm, a table feed speed of 39.37 mm per rotation, a pitch of 0.98, and a gantry rotation time of 0.5 s. Reconstructed images were displayed as 1.25 and 4 mm thick images. Both image sets were accessible to radiologists for image analysis.

All patients received i.v. injection of 600 mg iodine/Kg of nonionic iodinated contrast material at the concentration of 400 mg iodine/mL (Iomeron 400 Bracco SpA, Milan, Italy), using a commercially available power injector (CT-Injector Missouri XD 2001, Ulrich Medical, Ulm, Germany). After the acquisition of unenhanced images, a bolus-tracking program (Smart Prep; GE Healthcare, Milwaukee, WI, USA) was used to determine the time to initiate diagnostic scanning, after placing a circular Region of Interest in the aorta just above the diaphragmatic dome and fixing a threshold of 70 Hounsfield Units. When the bolus-tracking threshold was reached, triple-phase contrast-enhanced diagnostic scans were acquired with acquisition delays of 35 s for the arterial, 110 s for the venous, and 180 s for the equilibrium phases.

### 2.3. Fuzzy Logic Basic Principles and Comparison with the Theory of Probability

The notion of fuzzy set has been introduced by Zadeh [9] in order to formalize the concept of gradedness in class membership, in connection with the representation of human knowledge.

Fuzzy sets seem to be relevant in all the information-driven tasks where we need to make a classification based on data analysis and approximate reasoning. Gradedness in class membership is specified by the “membership function” *μ*_*F*_(*u*), which measures the degree of membership of an element *u* in a fuzzy set *F*, defined on a referential *U*. Literature provides at least three different interpretations of the concept of membership function, which are degree of similarity, preference, and uncertainty [12]. In the present study we used the semantics of uncertainty (in the general sense of incomplete knowledge, rather than only randomness), which is captured by fuzzy sets and fuzzy logics in the framework of possibility theory. This interpretation was proposed by Zadeh [13] when he introduced fuzzy logics, which is quite akin to “possibility theory,” and developed his theory of approximate reasoning [14]. *μ*_*F*_(*u*) is then the degree of possibility that a parameter *x* has value *u*, (the degree up to which the proposition “*x* = *u*” is true), given that all that is known about it is that “*x* is *F*” (e.g., “*x* is bald”). What happens is that the extreme values of the membership function are mutually exclusive, and the membership degrees rank these values in terms of their respective plausibility.

Uncertainty is often measured in terms of frequency of precise observed situations in a random experiment, and this approach leads to probability theory. However, uncertainty can also emerge in all cases in which it is not related with the frequency of random outcomes but with the imprecise nature of observations. When the repeated observations are precise, the probability assignments to the elements of *U* can be viewed as special membership functions such that the sum of membership grades is 1 (“singleton” fuzzy sets). On the other hand, when repeatedly observed situations are imprecise, more general kinds of membership functions are necessary. The degree of membership *μ*_*F*_(*u*) can then be computed as the proportion of observations that do not rule out the situation *u*. In this case, the membership function is interpreted as a “plausibility” function, since *μ*_*F*_(*u*) = 1 means that *u* is ruled out by no observation.

In the context of medical diagnosis, the degree of membership *μ*_*F*_(*u*), that is the proportion of observations that do not rule out the situation *u*, could be effectively measured (at least in principle) by the proportion of times the situation *u* has been tagged *F* in a random experiment where individuals presented to situation *u* are asked to put the tag “*F*” on *u* or not. This situation happens, for example, when (i) we show the same (blind) report to the same reader in different time, or (ii) we show much copies of the same (blind) report dispersed among a huge number of reports. Even if we are using relative frequencies, their mining is not that of probabilities, because no randomness is available, but rather a decision is relevant.

### 2.4. Fuzzy Logic Method and Image Analysis

Anonymized CT images from the venous phase were independently evaluated on a dedicated workstation (Osirix Aycan Workstation Osirix Pro, Rochester, NY, USA) by two radiologists with 15 (R1) and 5 (R2) years of experience in abdominal radiology, respectively. R1 and R2 were different radiologists than those who established final diagnoses by consensus. Both R1 and R2 were blinded to final diagnosis but not to the clinical status of patients, in order to reflect as much as possible the real clinical scenario. For the same purpose, R1 and R2 were left free to examine the whole liver in case of patients with multiple lesions. However, the visible CT examination was limited to the upper abdomen to avoid that eventual collateral findings might act as confounders.

To simplify the model, readers were aware that lesions were metastases or cysts at final diagnosis. Let *U* be a nonempty set indicating the universe of all possible test results and *P* and *N* two subsets of *U* containing the “positive test results” and “negative test results,” respectively. For each lesion, readers were asked to express their subjective DC in the positive diagnosis (*d*) of metastasis by means of the fuzzy term *P*(*d*); its value is included in the interval [0,1] (0 ≤ *P*(*d*) ≤ 1). Values were limited to the first decimal position, except for 0.51 and 0.49 indicating a *P*(*d*) near to the perfect uncertainty (0.50). In accordance with Castanho et al. [8], *P*(*d*) can be interpreted as the *membership degree *(or membership function) *μ*_*P*_(*d*) of a given radiological diagnosis to the fuzzy set *P*. By assuming a unitary value for the whole DC, the confidence in the alternative diagnosis of cysts (negative test result) will be

(1) $$\begin{array}{c} N\left(d\right)=1-P\left(d\right), \end{array}$$

corresponding to the complementary membership degree of that radiological diagnosis to the fuzzy set *N*. So *N*(*d*) can be interpreted as the *membership degree* *μ*_*N*_(*d*) of a given radiological diagnosis to the fuzzy set *N* [8].

In other words, we can express R1 and R2 diagnoses on a continuous interval as functions of DC, rather than on classical “crisp,” mutual exclusive basis 0 versus 1. Accordingly, a given diagnosis might belong at the same time to the sets *P* and *N* with complementary membership degrees. For example, a *P*(*d*_*i*_) = 0.8 for a given diagnosis *i* indicates that the lesion was interpreted as being a metastasis with a DC = 0.8 and a cyst with a DC = 0.2 (and vice versa for a *P*(*d*_*i*_) = 0.2).

### 2.5. Analysis of Readers' Diagnostic Performance

At the end of R1 and R2 readings, fifty couples of *P*(*d*) and *N*(*d*) values (one for each reading) were available. Analysis of the impact of DC on readers' performance was articulated in three steps. First, we cross-tabulated fuzzy data into a 2 × 2 table according to the results of the standards of reference. Because there is no graduation between a cyst and a metastasis, the latter was expressed on a crisp basis. In accordance with rules provided by Parasuraman et al. [15], the 2 × 2 table was interpreted as the result of the mathematical operation of aggregation, based on the min⁡⁡[*x*, *y*] operator, within the fuzzy subsets *P* and *N* (expressing the test results) and the subsets “test results associated with malignancy” (*M*) and “test results associated with benignancy” (*B*), that is,

(2) PM(x)=min⁡⁡[P(x),M(x)]PB(x)=P(x)−min⁡⁡[P(x),M(x)]NM(x)=N(x)−min⁡⁡[N(x),M(x)]NB(x)=min⁡⁡[N(x),B(x)].

Tables 1(a) and 1(b) illustrate how each couple of complementary fuzzy values was entered in the 2 × 2 table, according to the standard of reference result. Given all values, the resulting subsets *PM*, *PB*, *NM*, and *NB* corresponded, in the 2 × 2 table, to those of fuzzy true-positive (fTP), fuzzy false-positive (fFP), fuzzy true-negative (fTN), and fuzzy false-negative (fFN) cases, respectively. It has been demonstrated elsewhere [8] that global fTP, fTN, fFP, and fFN values correspond to the algebraic sum of complementary fuzzy membership degrees expressed for 30 patients with liver metastases (in the case of fTP and fFN diagnoses) and 20 patients with cysts (in the case of fFP and fTN diagnoses, resp.) (Table 2).

Second, we calculated per-lesion sensitivity, specificity, positive-predictive value (PPV), negative-predictive value (NPV), and accuracy for malignancy (together with 95% C.I.s) (i) on a crisp basis, that is, by assuming that 2 × 2 table cells were filled with mutual exclusive 1/0 values; (ii) on a fuzzy basis, that is, by using fTPs, fFNs, fFPs and fTNs resulting from the above method [8, 14]. Crisp diagnosis of malignancy or benignancy was assumed—for a given lesion—when *P*(*d*) or *N*(*d*) was equal to or larger than 0.51, respectively. In the case of perfect uncertainty (*P*(*d*) = 0.5), we assumed a crisp diagnosis of metastasis.

Finally, we assessed the impact of DC on diagnosis as the “divergence” *δ*(*F*, *C*) between fuzzy and crisp proportions, calculated as follows:

(3) $$\begin{array}{c} \delta \left(F,C\right)\left(\%\right)=\left(\frac{\text{crisp}\;\text{value}-\text{fuzzy}\;\text{value}}{\text{fuzzy}\;\text{value}}\right)\times 100. \end{array}$$

In the classical crisp approach for diagnosis, the reader is forced to press his own uncertainty (i.e., fuzzy DC levels) on a “flat” dichotomic basis 0-1. This leads to a skewed building of the 2 × 2 table based on the standard of reference. So the divergence *δ*(*F*, *C*) is here assumed to represent the “error” inherent to crisp values of diagnostic performance. In other words, *δ*(*F*, *C*) measures how much crisp evaluation differs from “the real state” of uncertainty (unexpressed in crisp setting) with which radiologists achieve a diagnosis, that is, the “real” diagnostic performance as adjusted for DC levels.

### 2.6. Simulations

We supposed that one reader (named R3) had examined CT images, showing crisp sensitivity, specificity, PPV, and NPV 66.7% (20/30), 50.0% (10/20), 66.7% (20/30), and 50.0% (10/10), respectively (together with binomial exact 95% C.Is). DC for correct diagnoses was assumed to be high, corresponding to *P*(*d*) of 0.9 for TPs. On the contrary, DC for incorrect diagnoses was simulated as follows: (i) scenario 1, in which R3 showed high DC levels, with *P*(*d*) of 0.9 for FPs; (ii) scenario 2, in which R3 showed lower DC levels, with *P*(*d*) of 0.7 for FPs.

Based on simulated membership degrees, we estimated fuzzy sensitivity, specificity, PPV, NPV, and accuracy (on a per-lesion basis) for both scenarios, together with the divergence *δ*(*F*, *C*) between crisp and fuzzy proportions.

## 3. Results

### 3.1. Readers' Diagnostic Performance

According to the crisp evaluation of CT images, R1 and R2 had 27 and 28 TPs, 3 and 2 FNs, 0 and 1 FPs, and 20 and 19 TNs, respectively. The set of readers' fuzzy diagnostic attributions are shown in Table 3, whereas 2 × 2 tables built on fuzzy membership degrees of corresponding fuzzy subsets are shown in Table 4(a) for R1 and Table 4(b) for R2. In the case of 30 patients with liver metastases, R1 achieved the same number of crisp and fuzzy TP and FN diagnoses (27 and 3, resp.). This was related to the high DC with which R1 made the majority of fuzzy diagnoses. In particular, *P*(*d*) and *N*(*d*) ranged from 0.8 to 1 in 27 and 2 patients, respectively (Table 4(a)). Concerning R2, (s)he was quite confident both in the majority of correct diagnoses (28/30 with *P*(*d*) = 1) and one incorrect diagnosis (*N*(*d*) = 1). However, since *P*(*d*) for the one FN diagnosis was 0.2 (i.e., *N*(*d*) = 0.8), fTPs (28.2) and fFNs (1.8) results slightly increased and decreased compared to crisp ones, that is, 28.2 and 1.8 versus 28 and 2, respectively (Table 4(b)).

In the case of patients with cysts, R1 attributed a *P*(*d*) = 0 (i.e., *N*(*d*) = 1) to 19 of 20 of them, but (s)he was less confident in one single diagnosis of benignancy (*P*(*d*) = 0.2). Compared to the crisp scenario, the effect was to slightly increase the FPs (from 0 to 0.2) and decrease the TNs (from 20 to 19.8) (Table 4). R2 made 12 TN and 1 FP diagnoses with maximal DC (*P*(*d*) = 0, i.e., *N*(*d*) = 1). Concerning the remaining 7 diagnoses, *P*(*d*) ranged between 0.1 and 0.3, leading to an increase in FPs (from 1 to 2.4) and a decrease in TN (from 19 to 17.6) compared to the crisp setting (Table 4(b)).

When estimating sensitivity, specificity, PPV, and NPV with the above fuzzy values, variations with respect to crisp proportions occurred as shown in Table 5. *δ*(*F*, *C*) (%) values are reported as well. In the case of R1 crisp diagnostic performance was slightly “overestimated” as compared to fuzzy ones in terms of specificity, PPV, NPV, and accuracy. In other words, positive *δ*(*F*, *C*) (%) values indicate that crisp performance was higher than that adjusted for the level of DC, that is, when R1 was not forced to express diagnoses dichotomically. The effect of DC level was more complex for R2, showing that the crisp performance was (i) slightly “underestimated” in the case of sensitivity and NPV and (ii) more largely overestimated in the case of the remaining estimates, especially specificity (*δ*(*C*, *F*) = +7.95%).

Difference between crisp and fuzzy proportions was not statistically significant (using an alfa of 0.05), as arguable from the overlap in 95% C.I.s.

### 3.2. Simulations

Results of simulations are shown in Table 6. According to the first scenario, confidently incorrect diagnoses contributed to increasing crisp diagnostic performance compared to the fuzzy one, except for specificity (*δ*(*F*, *C*)  (%) = 0).

On the contrary, lower level of DC in incorrect diagnoses led to a decrease of crisp performance in the second scenario (*δ*(*F*, *C*) (%) as low as −16.7% in the case of specificity). It is easy to demonstrate that *δ*(*F*, *C*) might have assumed a positive sign by entering less incorrect diagnoses in the model.

Difference between crisp and fuzzy proportions was not statistically significant (using an alfa of 0.05), as arguable from the overlap in 95% C.I.s.

## 4. Discussion

Fuzzy logic has been used in medicine to define complex epidemiological and clinical models for health decisions or risk prediction [16]. Applications in radiology have been mainly focused to test algorithms of image elaboration [17] or computer-aided diagnosis accounting for uncertainty inherent to lesions variability [18], as well as to fuzzify image findings features in the attempt to provide more effective diagnoses [19]. In the case of clinical applications, the basic principle has been to define to which extent one or more radiological features belonged to different diagnoses. Of these, the one showing radiological features with the highest membership degree is assumed to represent the “true” diagnosis. In summary, fuzzy logic has been applied as an instrument to facilitate diagnostic reasoning, whereas the fuzziness underlying the performance of a diagnostic test has been poorly investigated [20]. We used the method proposed by Castanho et al. [8] to express diagnoses on a fuzzy rather than binary base, thus estimating fuzzy sensitivity, specificity, NPV, PPV, and accuracy values. Differently from these authors, we investigate an imaging modality involving radiologists' decisions rather than objective results of a diagnostic tool. Accordingly, fuzzification was performed by using levels of DC as a measure of readers' uncertainty. One could argue that this choice is arbitrary and/or inappropriate. However, linguistic variables underlying fuzzy sets are a matter of interpretation [12], and our choice was founded on a mathematically consistent model, regardless of specific semantic attributions and specific causes of readers' uncertainty [8, 15]. It might be speculated that this is a potential advantage of this method, since uncertainty can be evaluated regardless of its origin (e.g., before and after an educational program or in different environmental conditions affecting readings). It should be pointed that, compared to previous works we presented in this field at European and American radiological congresses under the form of oral presentation [21] or posters ((1) Girometti et al., Electronic Posters, EPOS, European Congress of Radiology, Vienna 2013, doi: 10.1594/ecr2013/C-1495; (2) Girometti et al., Radiological Society of North America 98th Scientific Assembly and Annual Meeting, Chicago, 2012), the present paper refines the rules of diagnostic fuzzification according to the method proposed by Castanho et al. [8], providing novel and more robust results.

Given the above premises, we asked two radiologists to quantify the diagnosis of liver metastasis as a whichever value *P*(*d*) ranging from 0 to 1, based on the degree of DC at a single-time reading. A simplified clinical model was set for this purpose, providing one complementary alternative only (*N*(*d*) = 1 − *P*(*d*)) to main diagnosis (hepatic cyst versus metastasis at MDCT, resp.). Both radiologists were shown to be highly confident in making diagnoses, regardless of their correctness: they used *P*(*d*)/*N*(*d*) values of 1 or 0 in the majority of cases (44/50 and 40/50 lesions for R1 and R2, resp.). The degree of DC was relatively high even in more uncertain cases, corresponding to *P*(*d*)/*N*(*d*) no less than 0.8 for R1 and 0.7 for R2. Moreover, R2 showed average lower DC compared to R1, as expected on the basis of differential effects of experience (5 versus 15 years, resp.) [22]. In summary, we showed that differences in levels of DC have a measurable effect on the nominal, crisp diagnostic performance of radiologists, and that experience has a key role in such a determination. Fuzzy evaluation could be then used to define whether readers involved in radiological studies fulfil ideal criteria for image interpretation, that is, making correct diagnoses with high DC.

DC has been advocated to impact on diagnostic efficacy [2]. It is reasonable to assume that readers' efficacy will depend on how informative a diagnosis is, that is, on how much diagnostic “truth” it contains in order to guide the clinical workup. Given the uncertainty that unavoidably affects subjective image interpretation, we assumed that crisp diagnostic performance is not entitled to fully represent such a “truth,” that is, fuzzy diagnosis only is really representative of the “real state” of information carried by a diagnosis. Thus, we tested readers' efficacy by estimating the divergence *δ*(*F*, *C*) between fuzzy and crisp sensitivity, specificity, PPV, and NPV. In other words, we measured how much crisp performance is affected by levels of DC underlying radiological diagnoses, that is, how much information do diagnoses carry in the light of their subjective origin. Positive *δ*(*F*, *C*) values indicate that crisp diagnoses are overestimated as compared to fuzzy ones, because of lower levels of DC. In the case-study, R2 showed larger positive *δ*(*F*, *C*) (up to +7.95% in the case of specificity) compared to R1. One can suppose that a clinician aware of *δ*(*F*, *C*) values would probably find R1 diagnoses more informative than R2 ones and would probably change (or not) patients management based on the level of DC underlying radiological diagnoses, for example, by adding (or not) further diagnostic procedures before any treatment. It should be pointed that the estimate of *δ*(*F*, *C*) refers to a set of readings given the “truth” established by a standard of reference, that is, to the body of evidence provided by one radiologist on a large series of readings, as for example, occurs in an experimental study. The effect on the referring physician of expressing each single diagnosis as a fuzzy value in clinical practice, where the “truth” about the patient just comes at the end of the work-up process, should be a matter of a specific study, and its estimate was beyond the purpose of our work. In the case of negative values, *δ*(*F*, *C*) expresses how much crisp diagnosis is underestimated as compared to fuzzy one. This occurs when incorrect diagnoses are expressed with lower levels of DC, as shown in the case of R2 in the clinical setting. Since *N*(*d*) for one FN negative diagnosis was 0.8, the number of fNs slightly decreased compared to crisp ones (from 2 to 1.8, resp.). Accordingly, *δ*(*F*, *C*) for sensitivity and NPV was −0.71 and −0.27, respectively. This effect was emphasized by supposing more incorrect diagnoses with low DC, as we did in the second simulation. The diagnostic performance of the simulated reader (R3) was systematically lower on a crisp rather than a fuzzy basis, that is, when accounting for DC levels (up to −16.67% in the case of specificity). Thus, in a context of low accuracy readers should be unconfident in incorrect diagnoses in order to be more efficacious. On the other hand, high DC in incorrect diagnoses in the first simulation had the main effect to increase diagnostic accuracy (up to 5.26% in the case of sensitivity). In other words, DC level and *δ*(*F*, *C*) are able to communicate how confidently wrong diagnoses impact readers performance, leading to potential consequences in terms of clinical practice or evaluation of study results. Finally, *δ*(*F*, *C*), equal to zero when the algebraic sum of fuzzy and crisp elements in the 2 × 2 table is equivalent, as occurred for TP/fTP and TN/fTN cases of R1 in the clinical scenario (crisp and fuzzy sensitivities of 90.0% each) and FP/fFP and TN/fTN cases of R3 in the first simulation (crisp and fuzzy sensitivity of 50.0%). *δ*(*F*, *C*) might be zero also in the case readers would express all correct and incorrect diagnoses with *P*(*d*) = 1 and *N*(*d*) = 0, that is, in the case fuzzy membership degrees would correspond to crisp ones. We did not observe this result in our clinical series, confirming that readings of an experimental set are unavoidably affected by uncertainty, even if low. Accordingly, fuzzy diagnosis is expected to be more informative than crisp one.

Our work has some limitations. First, although differential diagnosis between small cysts and metastasis is relatively easy during imaging and clinical followup of oncologic patients, no histological examination was available to establish final diagnosis. However, general assumptions and applicability of our model do not depend on the presence of incorrect final diagnoses and subsequent variations in *δ*(*F*, *C*) potentially related to our series. Of note, we did not aim to test diagnostic accuracy of fuzzy evaluations per se but rather to verify whether effects of uncertainty are measurable in terms of *δ*(*F*, *C*). For this purpose, we used a simple, dichotomic model, regardless of its clinical importance. It is reasonable to infer that differences between crisp and fuzzy sensitivity, specificity, predictive values, and accuracy would be larger and statically significant in the case of more complex clinical scenario (e.g., screening mammography). Nonetheless, the absence of statistical significance does not imply a lack of clinical significance, as arguable from large *δ*(*F*, *C*) values in R3 simulations. Second, one might argue that two readers only might have been insufficient to achieve adequate statistical power in our analysis. However, we did not mean to estimate a somewhat variant of inter- or intrareader agreement and to establish how precise the estimate was. Divergence consists in the algebraic difference between crisp and fuzzy diagnostic performance as determined by readers' DC (see above); thus statistical power has no influence in its determination. Third, we gave emphasis to the concept of *δ*(*F*, *C*) in this study. One might argue that area under the curve (AUC) at receiver operating characteristic (ROC) analysis is more helpful in assessing uncertainty than *δ*(*F*, *C*). However, the role for fuzzy ROC curves in refining crisp evaluations was investigated elsewhere [8]. Our aim was to preliminarily determine whether (a) the method proposed by Castanho et al. [8] is applicable to real readers; (b) fuzzy and crisp performances are comparable in clinical scenario involving human readers.

In conclusion, we tested a fuzzy logic-based method accounting for readers' uncertainty (DC levels) on a real, simplified clinical scenario and two simulations using CT. According to this approach, a diagnosis can belong to positive or negative test results at the same time, differently from what occurs when considering diagnosis with binary (i.e., crisp) logic. By operating with the 2 × 2 table cells as fuzzy subsets, we calculated the divergence *δ*(*F*, *C*) between fuzzy and crisp diagnostic accuracy as a measure of the effect of DC levels on the nominal, crisp diagnostic performance (i.e., as a measure of diagnostic information). Potential applications of our method are in the context of clinical practice and/or experimental studies, and should be a matter for targeted investigation: first, by adjusting test results for readers' subjective interpretation; second, by testing the efficacy of readers involved in a study; third, by showing whether confidently incorrect diagnoses significantly affect readers' interpretation and fourth, by evaluating how training or educational programs improve the knowledge of less experienced radiologists. We expect that major application for this method would be—at least initially—in the setting of experimental studies. From the practical point of view, the estimate of *δ*(*F*, *C*) can be easily performed using a software program (see our website http://trm.twilightsubnet.com/fuzzy.php/). According to our results, ideal radiologists (and, by extension, a physician making a diagnosis) should be accurate, highly confident in correct diagnoses, and highly unconfident in incorrect diagnoses, leading to a *δ*(*C*, *F*) close to zero.

## Figures and Tables

**Table:** (a)

	*M*(*d*) = 1	*B*(*d*) = 0
*P*(*d*) = 0.7	min(0.7, 1) = 0.7	0.7 − [min(0.7, 1)] = 0
*N*(*d*) = 0.3	0.3 − [min(0.3, 0)] = 0.3	min(0.3, 0) = 0

**Table:** (b)

	*M*(*d*) = 0	*B*(*d*) = 1
*P*(*d*) = 0.8	min(0.8, 0) = 0	0.8 − [min(0.8, 0)] = 0.8
*N*(*d*) = 0.2	0.2 − [min(0.2, 1)] = 0	min(0.2, 1) = 0.2

*P*(*d*): fuzzy diagnosis of malignancy; *N*(*d*): fuzzy diagnosis of benignancy; *M*(*d*): standard of reference diagnosis of malignancy; *B*(*d*): standard of reference diagnosis of benignancy.

**Table 2:** Formulas for determining the total number of fuzzy true positives (fTPs), fuzzy false negatives (fFNs), fuzzy false positives (fFPs) and fuzzy true negatives (fTNs) used to estimate fuzzy sensitivity, specificity, PPV, NPV, and accuracy. Of note, those value can not be integer numbers.

	*M*(*d*) = 1	*B*(*d*) = 1
*P*(*d*)	fTPs = *PM*(*d*_1_) + ⋯+*PM*(*d*_*n*_)	fFPs = *PB*(*d*_1_) + ⋯+*PB*(*d*_*n*_)
*N*(*d*)	fFNs = *NM*(*d*_1_)+⋯+*NM*(*d*_*n*_)	fTNs = *NB*(*d*_1_) + ⋯+*NB*(*d*_*n*_)

**Table:** (a) R1 reader

Number of lesions	*P*(*d*)	*N*(*d*)
	Metastases	
25	1	0.0
2	0.8	0.2
2	0.2	0.8
1	0.0	1.0

	Cysts	
19	0.0	1.0
1	0.2	0.8

fTPs = (25 × 1.0)+(2 × 0.8)+(2 × 0.2)+(1 × 0.0) = 27

fFPs = (19 × 0.0)+(1 × 0.2) = 0.2

fFNs = (25 × 0.0)+(2 × 0.2)+(2 × 0.8)+(1 × 1.0) = 3

fTNs = (19 × 1.0)+(1 × 0.8) = 19.8

**Table:** (b) R2 reader

Number of lesions	*P*(*d*)	*N*(*d*)
	Metastases	
28	1	0.0
1	0.2	0.8
1	0.0	1.0

	Cysts	
12	0.0	1.0
2	0.1	0.9
3	0.2	0.8
2	0.3	0.7
1	1.0	0.0

fTPs = (28 × 1.0)+(1 × 0.2)+(1 × 0.0) = 28.2

fFPs = (12 × 0.0)+(2 × 0.1)+(3 × 0.2)+(2 × 0.3)+(1 × 1.0) = 2.4

fFNs = (28 × 0.0)+(1 × 0.8)+(1 × 1.0) = 1.8

fTNs = (12 × 1.0)+(2 × 0.9)+(3 × 0.8)+(2 × 0.7)+(1 × 0.0) = 17.6

fTPs: fuzzy true positives; fFNs: fuzzy false negatives; fFPs: fuzzy false positives; fTNs: fuzzy true negatives.

**Table:** (a)

R1	Standard of reference		Total
	Metastases	Cysts	
*P*(*d*)	27 (27)	0.2 (0)	27.2 (27)
*N*(*d*)	3 (3)	19.8 (20)	22.8 (23)
Total	**30 (30)**	**20 (20)**	**50**

**Table:** (b)

R2	Standard of reference		Total
	Metastases	Cysts	
*P*(*d*)	28.2 (28)	2.4 (1)	30.6 (29)
*N*(*d*)	1.8 (2)	17.6 (19)	19.4 (21)
Total	**30 (30)**	**20 (20)**	**50**

**Table 5:** Diagnostic accuracy of reader R1 and reader R2 in assessing malignancy of small focal liver lesions using multidetector row Computed Tomography (metastases versus cysts). Crisp and fuzzy accuracies are reported, together with 95% C.I.s (in brackets) and the divergence *δ*(*F*, *C*) (see the text).

	R1			R2		
	Crisp	Fuzzy	*δ*(*F*, *C*) (*%*)	Crisp	Fuzzy	*δ*(*F*, *C*) (*%*)
Sensitivity (%)	90.0	90.0		93.3	94.0	
	(77.4–96.3)	(77.4–96.3)	0	(81.6–98.1)	(82.5–98.4)	−0.71
	(27/30)	(27/30)		(28/30)	(28.2/30)	

Specificity (%)	100	99.0		95.0	88.0	
	(91.1–100)	(89.5–100)	+1.01	(83.8–98.9)	(75.0–95.0)	+7.95
	(20/20)	(19.8/20)		(19/20)	(17.6/20)	

PPV (%)	100	99.3		96.6	92.2	
	(91.1–100)	(89.9–100)	+0.74	(85.9–99.5)	(80.1–97.5)	+4.77
	(27/27)	(27/27.2)		(28/29)	(28.2/30.6)	

NPV (%)	87.0	86.8		90.5	90.7	
	(73.3–94.4)	(73.6–94.3)	+0.13	(78.0–96.5)	(78.3–96.7)	−0.27
	(20/23)	(19.8/22.8)		(19/21)	(17.6/19.4)	

Accuracy (%)	94.0	93.6		94.0	91.6	
	(82.5–98.4)	(81.9–98.2)	+0.43	(82.5–98.4)	(79.4–97.2)	+2.62
	(47/50)	(46.8/50)		(47/50)	(45.8/50)	

**Table 6:** Fuzzy performance of a simulated reader R3 showing low crisp accuracy for malignancy (metastases versus cysts at multidetector row Computed Tomography) and high diagnostic confidence (DC) in correct diagnoses. Simulations 1 and 2 assume that DC in incorrect diagnoses (false negatives and false positives) are expressed with high or low DC levels, respectively (see the text for further detail). In brackets we reported 95% C.I.s.

	Crisp	Simulation 1		Simulation 2	
		Fuzzy	*δ*(*F*, *C*) (%)	Fuzzy	*δ*(*F*, *C*) (%)
Sensitivity (%)	66.7	63.3		70.0	
	(51.8–79.0)	(48.5–76.1)	+5.26	(55.2–81.7)	−4.76
	(20/30)	(19/30)		(21/30)	

Specificity (%)	50.0	50.0		60.0	
	(35.7–64.3)	(35.7–64.3)	0	(54.2–73.3)	−16.7
	(10/20)	(10/20)		(12/20)	

PPV (%)	66.7	65.5		72.4	
	(51.8–79.0)	(50.7–78.0)	+1.75	(57.7–83.7)	−7.94
	(20/30)	(19/29)		(21/29)	

NPV (%)	50.0	47.6		57.1	
	(35.7–64.3)	(33.5–62.1)	+5.00	(42.4–70.8)	−12.5
	(10/20)	(10/21)		(12/21)	

Accuracy (%)	60.0	58.0		66.0	
	(54.2–73.3)	(43.3–71.5)	+3.45	(51.1–78.4)	−9.09
	(30/50)	(29/50)		(33/50)	
